# Venous Thromboembolism Chemoprophylaxis Adherence Rates After Major Cancer Surgery

**DOI:** 10.1001/jamanetworkopen.2023.35311

**Published:** 2023-09-28

**Authors:** Charles D. Logan, Matthew T. Hudnall, Cary Jo R. Schlick, Dustin D. French, Brian Bartle, Dominic Vitello, Hiten D. Patel, Lauren M. Woldanski, Daniel E. Abbott, Ryan P. Merkow, David D. Odell, David J. Bentrem

**Affiliations:** 1 Northwestern Quality Improvement, Research, & Education in Surgery, Department of Surgery, Northwestern University, Feinberg School of Medicine, Chicago, Illinois; 2 Canning Thoracic Institute, Department of Surgery, Northwestern University, Feinberg School of Medicine, Chicago, Illinois; 3 Surgery Service, Jesse Brown VA Medical Center, Chicago, Illinois; 4 Department of Urology, Northwestern University, Feinberg School of Medicine, Chicago, Illinois; 5 Department of Ophthalmology, Northwestern University, Chicago, Illinois; 6 Center for Health Services and Outcomes Research, Northwestern University, Chicago, Illinois; 7 Veterans Affairs Health Services Research and Development Service, Chicago, Illinois; 8 Department of Medical Social Sciences, Northwestern University, Chicago, Illinois; 9 US Department of Veterans Affairs, Center of Innovation for Complex Chronic Healthcare, Hines VA Medical Center, Chicago, Illinois; 10 Department of Surgery, University of Wisconsin School of Medicine and Public Health, Madison; 11 William S. Middleton VA Medical Center, Madison, Wisconsin

## Abstract

**Importance:**

Venous thromboembolism (VTE) represents a major source of preventable morbidity and mortality and is a leading cause of death in the US after cancer surgery. Previous research demonstrated variability in VTE chemoprophylaxis prescribing, although it is unknown how these rates compare with performance in the Veterans Health Administration (VHA).

**Objective:**

To determine VTE rates after cancer surgery, as well as rates of inpatient and outpatient (posthospital discharge) chemoprophylaxis adherence within the VHA.

**Design, Setting, and Participants:**

This retrospective cohort study within 101 hospitals of the VHA health system included patients aged 41 years or older without preexisting bleeding disorders or anticoagulation usage who underwent surgical treatment for cancer with general surgery, thoracic surgery, or urology between January 1, 2015, and December 31, 2022. The VHA Corporate Data Warehouse, Pharmacy Benefits Management database, and the Veterans Affairs Surgical Quality Improvement Program database were used to identify eligible patients. Data analysis was conducted between January 2022 and July 2023.

**Exposures:**

Inpatient surgery for cancer with general surgery, thoracic surgery, or urology.

**Main Outcomes and Measures:**

Rates of postoperative VTE events within 30 days of surgery and VTE chemoprophylaxis adherence were determined. Multivariable Poisson regression was used to determine incidence-rate ratios of inpatient and postdischarge chemoprophylaxis adherence by surgical specialty.

**Results:**

Overall, 30 039 veterans (median [IQR] age, 67 [62-71] years; 29 386 men [97.8%]; 7771 African American or Black patients [25.9%]) who underwent surgery for cancer and were at highest risk for VTE were included. The overall postoperative VTE rate was 1.3% (385 patients) with 199 patients (0.7%) receiving a diagnosis during inpatient hospitalization and 186 patients (0.6%) receiving a diagnosis postdischarge. Inpatient chemoprophylaxis was ordered for 24 139 patients (80.4%). Inpatient chemoprophylaxis ordering rates were highest for patients who underwent procedures with general surgery (10 102 of 10 301 patients [98.1%]) and lowest for patients who underwent procedures with urology (11 471 of 17 089 patients [67.1%]). Overall, 3142 patients (10.5%) received postdischarge chemoprophylaxis, with notable variation by specialty.

**Conclusions and Relevance:**

These findings indicate the overall VTE rate after cancer surgery within the VHA is low, VHA inpatient chemoprophylaxis rates are high, and postdischarge VTE chemoprophylaxis prescribing is similar to that of non-VHA health systems. Specialty and procedure variation exists for chemoprophylaxis and may be justified given the low risks of overall and postdischarge VTE.

## Key Points

**Question:**

What are the rates of venous thromboembolism (VTE) diagnosis and postoperative chemoprophylaxis adherence following major cancer surgery within the Veterans Health Administration?

**Findings:**

In this cohort study including 30 039 veterans surgically treated for cancer, the overall rate of VTE rates was 1.3%, with variations in chemoprophylaxis ordering rates by surgical specialty and procedure site.

**Meaning:**

These findings suggest that there is variation in chemoprophylaxis by specialty and procedure site within the Veterans Health Administration, which may be justified given the low risk of overall VTE and postdischarge VTE.

## Teaser

This cohort study assesses the rate of venous thromboembolism after cancer surgery and the rate of chemoprophylaxis adherence within the Veterans Health Administration.

## Introduction

Venous thromboembolism (VTE), which includes deep vein thrombosis (DVT) and pulmonary embolism (PE), is a common complication associated with hospitalization and remains a major cause of excessive length of hospital stay and mortality.^1^ VTE is the leading cause of perioperative mortality after major cancer surgery, and an abundance of evidence shows that use of both inpatient and postdischarge chemoprophylaxis can reduce the risk of VTE.^2,3,4,5,6^ Established guidelines recommend extended VTE chemoprophylaxis postdischarge for up to 4 weeks after major surgery for abdominal or pelvic cancer.^7,8,9,10^ In 2021, the American Association for Thoracic Surgery and the European Society for Thoracic Surgery jointly established the first guidelines recommending extended VTE chemoprophylaxis postdischarge for 28 to 35 days for patients undergoing surgery of the lung and esophagus.^11,12,13^

Recent studies^14,15^ have shown high concordance with postsurgery inpatient chemoprophylaxis guidelines but lower concordance for outpatient postdischarge chemoprophylaxis. Yang et al^14^ found that more than 80% of inpatients received chemoprophylaxis after major surgery in the Illinois surgical collaborative. Similarly, in a single-center analysis, Ramanathan et al^15^ found more than 80% compliance for inpatient chemoprophylaxis, with noted variability among surgical specialties. Even more variability has been shown in outpatient ordering of chemoprophylaxis after major cancer surgery, with rates ranging from 1% to 30%.^2,16,17^

However, it is unknown how VTE and chemoprophylaxis rates reported in previous studies compare with those in large health systems such as the Veterans Health Administration (VHA). Our hypothesis was that the VHA would have similar rates of VTE and VTE chemoprophylaxis inpatient ordering and postdischarge prescribing among patients undergoing elective inpatient surgery for cancer compared with non-VHA hospitals, with variation by surgical specialty and procedure site. Therefore, the objective of this study was to determine VTE rates after major cancer surgery, as well as rates of chemoprophylaxis inpatient ordering and postdischarge prescribing within the VHA by surgical specialty and procedure site.

## Methods

### Data Source and Population

This cohort study followed the Strengthening the Reporting of Observational Studies in Epidemiology (STROBE) reporting guideline,^18^ and was approved by the Jesse Brown VA Medical Center institutional review board with a waiver of informed consent because data were deidentified in accordance with 45 CFR§46. The VHA Corporate Data Warehouse (VHA-CDW) and Veterans Affairs Surgical Quality Improvement Program (VASQIP) database were used to identify all patients who underwent a major inpatient surgery for cancer within the VHA with general surgery, thoracic surgery, or urology between January 1, 2015, and December 31, 2022 (eTables 1-3 in Supplement 1). The VHA-CDW contains electronic medical records from the Veterans Health Information Systems and Technology Architecture. The VHA-CDW also contains pharmacy and laboratory values. Encrypted patient identifiers enable linkages between data sources, including the VASQIP database and the Pharmacy Benefits Management Services database. Patients with preexisting bleeding disorders (1710 patients) or history of VTE (1783 patients) and patients who were taking anticoagulation or antiplatelet therapy preoperatively (2374 patients) were excluded. Additionally, 27 patients with missing height and weight data, 179 patients with unknown type of surgical procedure, 795 patients with missing operative time, 340 patients who underwent procedures with operative time of less than 45 minutes, 260 patients aged 40 years or younger, and 3055 who were discharged on postoperative day 0 or with missing length of stay were excluded.

### Patient Characteristics

VASQIP provided patient-specific variables related to age, sex, race and ethnicity, body mass index (calculated as weight in kilograms divided by height in meters squared), functional status, preoperative comorbidities, and postoperative complications. Age was categorized similarly according to the Caprini VTE Risk Calculator^19,20^ into 40 years and younger (excluded from our analysis), 41 to 60, 61 to 74, and 75 years or older. Race and ethnicity were determined by self-report and categorized as African American or Black, American Indian or Alaska Native, Asian or Pacific Islander, Hispanic, non-Hispanic White, and other or unknown race or ethnicity (defined as patients who declined to answer and those who indicated unknown). Reporting of race and ethnicity in this study was included to be consistent with policies set forth by the US National Institutes of Health.^21^ Body mass index was categorized according to recognized categories of less than 18.5, 18.5 to less than 25, 25 to less than 30, and greater than or equal to 30. Functional status was categorized as independent vs dependent.

### Clinical and Facility Characteristics

Variables for the surgical procedure site (colorectal, hepatobiliary, pancreas, esophagogastric, esophagus, lung, kidney, bladder, and prostate), and the surgical specialty performing the procedure (general surgery, thoracic surgery, and urology) were considered. Operation duration was categorized into quartiles (0.75 to 2.9 hours, >2.9 to 3.9 hours, >3.9 to 5.1 hours, and >5.1 hours). VASQIP also provided specific variables related to regional designation, specific facility, and facility-level complexity scores.

### Outcomes

The dependent variable of interest was VTE chemoprophylaxis ordering or prescribing for inpatients and outpatients. Adherence was defined at a clinician level. Inpatient adherence was defined as ordering VTE chemoprophylaxis for postoperative inpatients. Postdischarge adherence was defined as prescribing VTE chemoprophylaxis for outpatient administration. Secondary outcomes included VTE inpatient events and VTE outpatient (posthospital discharge) events, including both DVT and PE, detected postoperatively within 30 days by VASQIP record review. Patient records pertaining to chemoprophylaxis ordering, administration, and prescribing data were merged with each patient in the VASQIP data set, including patients from 101 national VHA hospitals. Patient factors and outcomes were identified and matched from the VASQIP data set and VHA-CDW with information confirmed using Veterans Health Information Systems and Technology Architecture Inpatient Menus for Pharmacy, Pharmacy Benefits Management Services, as well as the Barcode Medication Administration records.

### Statistical Analysis

#### Descriptive Statistics

For continuous variables, medians and IQRs for nonnormally distributed variables and means and SDs for normally distributed variables were evaluated. Differences between medians were evaluated with Wilcoxon signed rank tests, whereas differences between means were evaluated with Student *t* tests as appropriate.

#### Bivariable Analysis

Pearson χ^2^ and Fisher exact tests were used as appropriate to determine the significance of differences between patient, clinical, and facility characteristics and overall VTE rates, as well as the proportion of VTEs diagnosed inpatient vs postdischarge. Rates of appropriate inpatient VTE chemoprophylaxis and discrepancies between doses ordered and doses given were evaluated. Rates of postdischarge DVT chemoprophylaxis prescribing were also determined. Further analysis by surgical specialty (general surgery, thoracic surgery, or urology) and procedure site were conducted. *P* values were 2-sided and were considered statistically significant at *P* < .05.

#### Multivariable Poisson Regression Modeling

Independent variables were chosen for the model if they were factors in VTE risk calculators or were previously known to be associated with VTE risk.^19,20,22^ Additional independent variables were those identified as significant in bivariable analysis and confirmed with Akaike information criterion model selection for fit. Multivariable Poisson regression was used to determine the association of surgical specialty (general surgery, thoracic surgery, and urology) with rates of inpatient VTE chemoprophylaxis ordering (model 1) and rates of postdischarge VTE chemoprophylaxis prescribing (model 2) while adjusting for age, sex, race and ethnicity, body mass index, operative time, postoperative length of stay, functional status, history of tobacco use, chronic obstructive pulmonary disease, dyspnea, cardiovascular disease, type 1 or type 2 diabetes, history of coronary artery disease, history of congestive heart failure, disseminated cancer, weight loss, or history of esophageal varices. Model SEs were adjusted for clustering within facilities. Results were reported with incidence-rate ratios and 95% CIs. All analyses were done with Stata MP statistical software version 17 (Stata Corp) from January 2022 to July 2023.

## Results

### Characteristics of the National Patient Cohort

After excluding 10 523 patients (25.9%), 30 039 patients (median [IQR] age, 67 [62-71] years; 29 386 men [97.8%]; 7771 African American or Black patients [25.9%]) treated at 101 VHA facilities met criteria for inclusion. All included patients were at highest risk for VTE and without a history of VTE.^19,20,23^ In total, 8859 patients (29.5%) had a history of cigarette smoking within 1 year of surgery. General surgery treated 10 301 patients (34.3%), thoracic surgery treated 2649 patients (8.8%), and urology treated 17 089 patients (56.9%) (Table 1). The most common procedure sites were prostate (11 357 patients [37.8%]), colorectal (7968 patients [26.5%]), kidney (4603 patients [15.3%]), and lung (2282 patients [7.6%]) (eTable 4 in Supplement 1). The median (IQR) postoperative hospital length of stay was 4 (2-7) days for the entire cohort. Median (IQR) postoperative hospital length of stay was 6 (4-8) days for colorectal procedures, 6 (4-8) days for hepatobiliary procedures, 9 (7-16) days for pancreas procedures, 9 (7-15) days for esophagogastric procedures, 12 (9-18) days for esophagus procedures, 7 (5-9) days for lung procedures, 3 (2-4) days for kidney procedures, 7 (6-11) days for bladder procedures, and 2 (1-3) days for prostate procedures (eTable 4 in Supplement 1).

**Table 1.** Nationwide Veterans Health Administration Cohort Patient Characteristics by Surgical Specialty

Parameter	Patients, No. (%)				*P* value
	Total (N = 30 039)	General surgery (n = 10 301)	Thoracic surgery (n = 2649)	Urology (n = 17 089)	
Age, y					
41-60	6896 (23.0)	2044 (19.8)	398 (15.0)	4454 (25.1)	<.001
60-74	19 361 (64.4)	6124 (59.4)	1888 (71.3)	11 349 (66.4)	
≥75	3782 (12.6)	2133 (20.7)	363 (13.7)	1286 (7.5)	
Sex					
Male	29 386 (97.8)	9911 (96.2)	2558 (96.6)	16 917 (99.0)	NA
Female	653 (2.2)	390 (3.8)	91 (3.4)	172 (1.0)	
Race and ethnicity					
African American or Black	7771 (25.9)	2143 (20.8)	431 (16.3)	5197 (30.4)	<.001
American Indian or Alaska Native	230 (0.8)	89 (0.9)	21 (0.8)	120 (0.7)	
Asian or Pacific Islander	358 (1.2)	137 (1.3)	26 (1.0)	195 (1.1)	
Hispanic	2024 (6.7)	657 (6.4)	123 (4.6)	1244 (7.3)	
Non-Hispanic White	17 899 (59.6)	6628 (64.3)	1902 (71.8)	9369 (54.8)	
Other or unknown^a^	1757 (5.8)	647 (6.3)	146 (5.5)	964 (5.6)	
Body mass index^b^					
Underweight (<18.5)	590 (2.0)	300 (2.9)	79 (3.0)	211 (1.2)	<.001
Normal (18.5 to <25)	7538 (25.1)	3003 (29.1)	882 (33.3)	3653 (21.4)	
Overweight (25 to <30)	11 109 (37.0)	3591 (34.9)	925 (34.9)	6593 (38.6)	
Obese (≥30)	10 802 (36.0)	3407 (33.1)	763 (28.8)	6632 (38.8)	
Operation duration quartiles, h					
0.75-2.9	7558 (25.1)	3740 (36.3)	771 (29.1)	3047 (17.8)	<.001
>2.9 to 3.9	7349 (24.5)	2241 (21.8)	728 (27.5)	4380 (25.6)	
>3.9 to 5.1	7385 (24.6)	1808 (17.5)	518 (19.5)	5059 (29.6)	
>5.1	7747 (25.8)	2512 (24.4)	632 (23.8)	4603 (26.9)	
Venous thromboembolism rates					
Overall	385 (1.3)	159 (1.5)	36 (1.4)	190 (1.1)	.008
Inpatient	199 (0.7)	110 (1.1)	24 (0.9)	65 (0.4)	<.001
Postdischarge	186 (0.6)	49 (0.5)	12 (0.4)	125 (0.7)	.02

Abbreviation: NA, not applicable.

^a^ Other or unknown race or ethnicity includes patients who declined to answer and patients who indicated unknown.

^b^ Body mass index is calculated as weight in kilograms divided by height in meters squared.

### Rates of VTE and Perioperative Mortality

In the entire cohort, 385 patients (1.3%) received a diagnosis of VTE, with 199 (0.7%) who received a diagnosis as an inpatient and 186 (0.6%) who received a diagnosis postdischarge (Table 1). Of the patients with VTE, 247 (64.1%) received a diagnosis of PE (123 inpatient and 124 postdischarge). Among 377 patients (1.3%) in the total cohort who died within 30 days of their surgical procedure, 22 patients (5.7%) had received a diagnosis of VTE (5 patients with DVT and 17 patients with PE). VTE was the fifth most common cause of perioperative mortality in this cohort.

Of the 10 301 patients who underwent general surgery, 159 (1.5%) received a diagnosis of VTE, with the majority being in the inpatient setting (110 patients [69.2%]) vs postdischarge setting (49 patients [30.8%]). Overall, 36 of the 2649 patients (1.4%) who underwent a thoracic surgery operation received a diagnosis of VTE, with 24 (66.7%) inpatient and 12 (33.3%) postdischarge. Additionally, of the 7089 patients who underwent a urology operation, 190 (1.1%) received a diagnosis of VTE, with fewer receiving diagnoses as inpatients (65 patients [34.3%]) vs postdischarge (125 patients [65.7%]).

**Figure 1.**
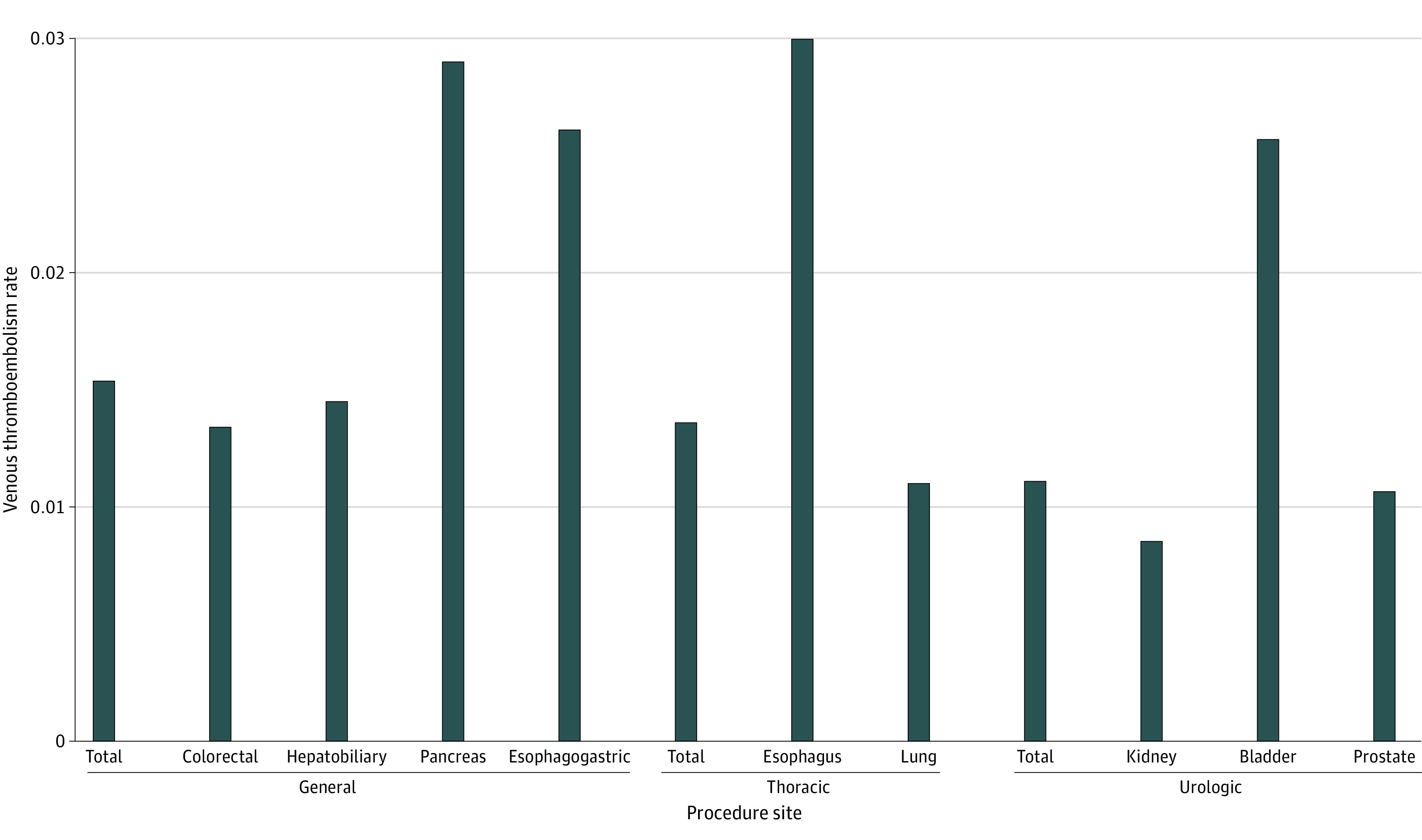
Venous Thromboembolism Rates by Surgical Specialty and Procedure Site Within the Veterans Health Administration The figure shows rates of venous thromboembolism by surgical specialty (general, thoracic, and urology) and procedure site. Overall rates of VTE were highest for patients who underwent esophagus, bladder, and pancreas procedures (P < .001).

The lowest rates of VTE by site were for patients who underwent procedures of the prostate (11 357 patients [1.1%]) and kidney (4603 patients [0.9%]) (eTable 4 in Supplement 1). Overall rates of VTE were highest for patients who underwent esophagus (367 patients [3.0%]), bladder (1129 patients [2.6%]), and pancreas (792 patients [2.9%]) procedures (Figure 1).

### Inpatient VTE Chemoprophylaxis by Specialty and Procedure

**Figure 2.**
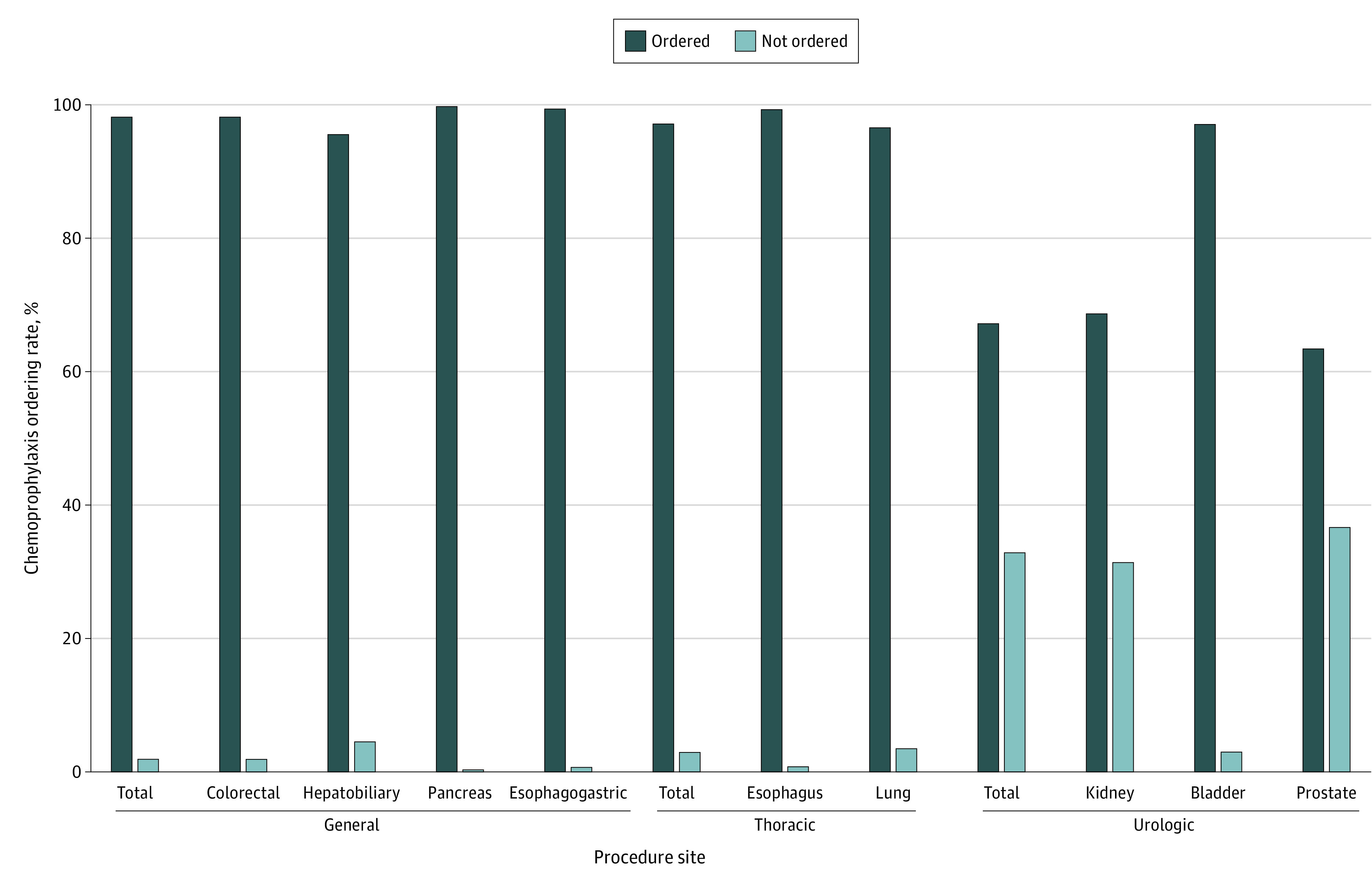
Inpatient Venous Thromboembolism Chemoprophylaxis Ordering by Surgical Specialty and Procedure Site Within the Veterans Health Administration The figure shows inpatient venous thromboembolism chemoprophylaxis ordering rates by surgical specialty (general, thoracic, and urology) and procedure site.

Overall, 24 139 patients (80.4%) received orders for inpatient DVT chemoprophylaxis (Table 2). Of the patients with orders for inpatient DVT chemoprophylaxis, 24 026 (99.5%) had at least 1 dose administered. General surgery had the highest rate of VTE chemoprophylaxis prescribing for inpatients (10 102 of 10 301 patients [98.1%]) compared with thoracic surgery (2566 of 2649 patients [96.9%]) and urology (11 471 of 17 089 patients [67.1%]) (Table 2 and Figure 2).

**Table 2.** Nationwide Veterans Health Administration Cohort Surgical Specialty Variation in Venous Thromboembolism Chemoprophylaxis After Major Cancer Surgery

Surgical specialty	Inpatient VTE chemoprophylaxis (model 1)		Postdischarge VTE chemoprophylaxis (model 2)^a^	
	Patients, No./total No. (%)^b^	IRR (95% CI)	Patients, No.total/No. (%)^b^	IRR (95% CI)
General surgery	10 102/10 301 (98.1)	1 [Reference]	1703/10 301 (16.5)	1 [Reference]
Thoracic surgery	2566/2649 (96.9)	0.96 (0.94-0.98)	76/2649 (2.9)^a^	0.16 (0.05-0.53)
Urology	11 471/17 089 (67.1)	0.69 (0.63-0.76)	1363/17 089 (8.0)	0.42 (0.24-0.72)
Overall	24 139/30 039 (80.4)	NA	3142/30 039 (10.5)	NA

Abbreviations: IRR, incidence-rate ratio; NA, not applicable.

^a^ Study period largely predates the 2021 American Association of Thoracic Surgery and European Society for Thoracic Surgery guidelines for cancer-associated venous thromboembolism in thoracic surgery.

^b^ All *P* < .001.

Patients who underwent surgical procedures of the prostate (11 357 patients [62.9%]) and kidney (4603 patients [68.3%]) had the lowest rates of inpatient VTE chemoprophylaxis ordering. Rates of inpatient VTE chemoprophylaxis ordering were similar for patients who underwent lung (2282 patients [96.4%]), esophagogastric (575 patients [98.1%]), esophagus (367 patients [99.2%]), colorectal (7968 patients [97.9%]), hepatobiliary (966 patients [95.3%]), pancreas (792 patients [99.7%]), and bladder (1129 patients [96.9%]) surgery (Figure 2).

### Postdischarge VTE Chemoprophylaxis by Specialty and Procedure

**Figure 3.**
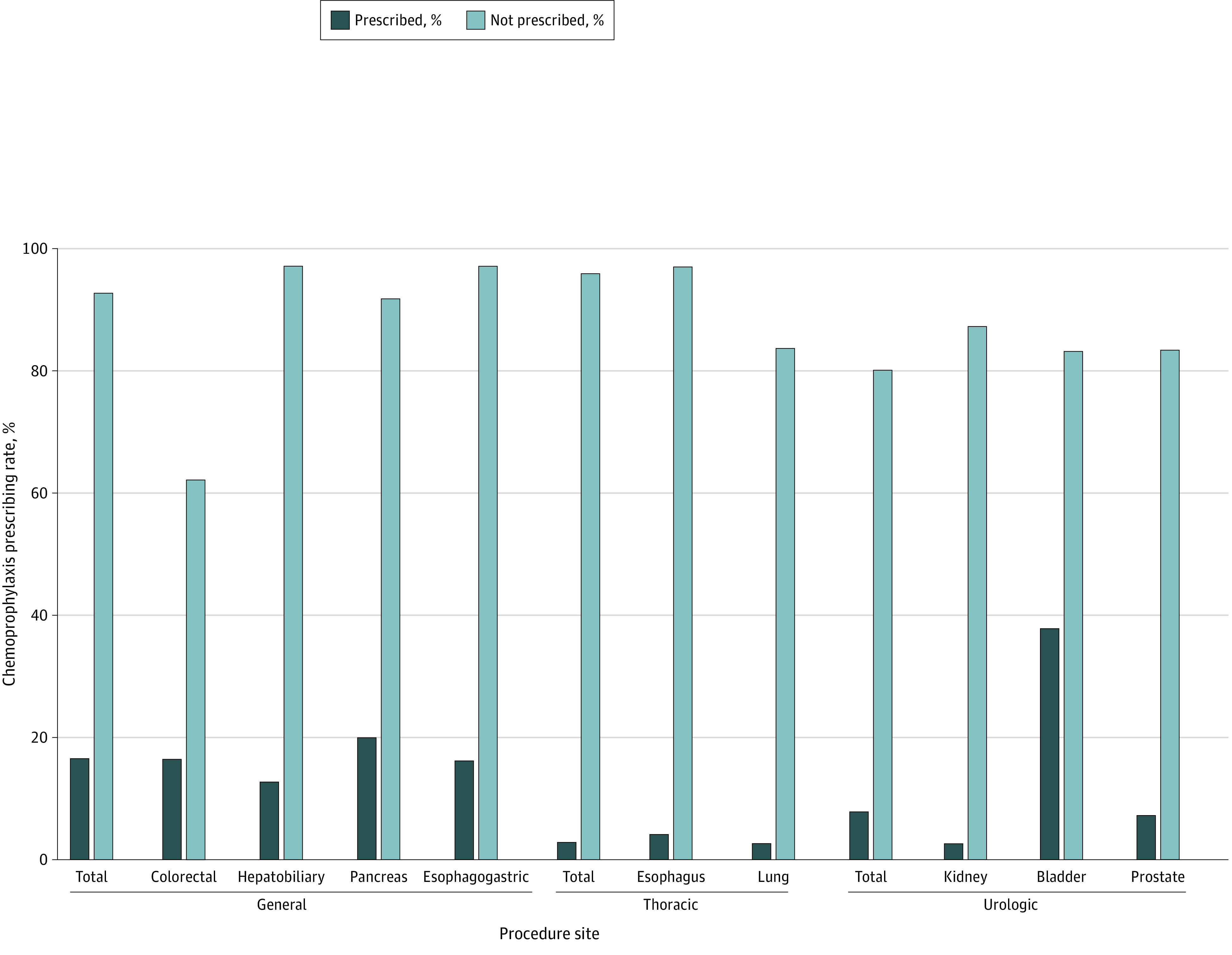
Postdischarge Venous Thromboembolism Chemoprophylaxis Prescribing by Surgical Specialty and Procedure Site Within the Veterans Health Administration The figure shows the postdischarge venous thromboembolism chemoprophylaxis prescribing rates by surgical specialty (general, thoracic, and urology) and procedure site. Postdischarge VTE chemoprophylaxis prescribing varied for patients who underwent colorectal, hepatobiliary, pancreas, esophagogastric, bladder, and prostate procedures (*P* < .001). The study period predates the 2021 American Association of Thoracic Surgery and European Society for Thoracic Surgery guidelines for cancer-associated venous thromboembolism in thoracic surgery.

Overall, 3142 patients (10.5%) received prescriptions for postdischarge DVT chemoprophylaxis. General surgery had the highest rate of VTE chemoprophylaxis prescribing for patients postdischarge (1703 of 10 301 patients [16.5%]) compared with thoracic surgery (76 of 2649 patients [2.9%]) and urology (1363 of 17 089 patients [8.0%]) (Table 2 and Figure 3).

Postdischarge VTE chemoprophylaxis prescribing rates were lowest for patients who underwent lung (2282 patients [2.7%]), esophagus (367 patients [4.1%]), and kidney (4603 patients [2.5%]) surgery. Postdischarge VTE chemoprophylaxis prescribing varied for patients who underwent colorectal (7968 patients [16.7%]), hepatobiliary (966 patients [12.6%]), pancreas (792 patients [19.9%]), esophagogastric (575 patients [16.2%]), bladder (1129 patients [37.6%]), and prostate (11 357 patients [7.2%]) surgery (Figure 3).

## Discussion

In this cohort study using VASQIP data reported by 101 VHA hospitals nationwide, we found that overall VTE rates for patients at the highest risk after major cancer surgery within the VHA were low (1.3%). VTE chemoprophylaxis adherence for inpatients was high for patients treated by general or thoracic surgery but lower for those treated by urology who underwent procedures of the kidney or prostate.

Previously, Holcomb et al^5^ found an overall VTE rate of 1.4% in the VHA with a study population including patients both with and without cancer, with a majority (57%) undergoing orthopedic procedures. In a National Surgical Quality Improvement Program study of patients with cancer, Merkow et al^17^ found the VTE rate to be 1.6% in a study population that consisted of patients with colorectal cancer (33%) and breast cancer (30%).

Despite variation by specialty with regard to inpatient VTE chemoprophylaxis ordering, rates of VTE in the VHA were similar to those in other non-VHA health systems.^17,22,24,25,26^ Inpatient VTE chemoprophylaxis administration is a multilevel process with many areas for potential failure, but we found that rates of ordering (80.4%) were concordant with rates of documented administration (99.5%), which indicates a comparably high level of performance at VHA hospitals.^14^

Postdischarge VTE chemoprophylaxis prescribing was similar to that in other non-VHA health systems and was low, with only 10.5% of patients nationally receiving a prescription. These findings represent an opportunity for improvement, although it is notable that postdischarge VTE chemoprophylaxis guidelines for lung surgery had not been established until late in this study period.^12^ However, given that our data show that variation in postdischarge VTE chemoprophylaxis prescribing was particularly notable, improvement initiatives appropriately targeted by specialty and procedure may be warranted. There may also be a need for individualized risk-based prescribing of postdischarge prophylaxis given the variation associated with procedure site.^24,25,27^

Some specialty variation for chemoprophylaxis may be justified given the low risks of overall VTE and postdischarge VTE. We found that patients who underwent urological procedures of the kidney and prostate were significantly less likely to receive inpatient VTE chemoprophylaxis compared with those who underwent any type of operation with general or thoracic surgery. Although patients who underwent prostate and kidney operations had the lowest rates of inpatient VTE chemoprophylaxis, rates of chemoprophylaxis for patients who underwent bladder operations were comparable to those for patients who underwent procedures with other specialties, and also had the highest rates of outpatient VTE chemoprophylaxis prescribing. This variation is likely due to practitioner preference and the perceived risk of bleeding after specific urological procedures balanced with the risk of VTE. Considering the overall national VTE rates for patients undergoing urological procedures of the kidney and prostate were 0.9% and 1.1%, respectively, the risks are likely being appropriately assessed at the practitioner level.

Because VTE chemoprophylaxis has been associated with positive outcomes, the majority of efforts have been focused on improving administration rates both for inpatient and outpatient procedures. Indeed, continued efforts to minimize missed doses of ordered inpatient VTE chemoprophylaxis are warranted for high-risk procedure sites due to the association with increased VTE rates among these patients.^28^ However, risk-based use of VTE chemoprophylaxis is less common, with some notable exceptions. These include the use of aspirin for chemoprophylaxis after orthopedic procedures and risk calculation to determine the need for extended postdischarge VTE chemoprophylaxis after bariatric surgery.^29,30^ This study suggests that risk-based deescalation may be warranted for certain urological operations, specifically those involving the prostate or kidney that have short lengths of hospital stay and high utilization of minimally invasive surgery. This study also supports further development of VTE risk calculators that are both population-specific and procedure site–specific to guide chemoprophylaxis use.^22,24,25^

### Limitations

Our study has several limitations, including a study population composed mostly of older men, which limits generalizability. Although we can comment with confidence on the low rates of prescribing of postdischarge VTE chemoprophylaxis in the VHA health system, actual outpatient VTE chemoprophylaxis rates of self-administration are unclear. Although patients receive training on administration of enoxaparin injections, there is a limitation in that there is no documentation of actual reported or measured patient home administration. It is possible that actual patient postdischarge VTE chemoprophylaxis is functionally lower than the low rates reported in this study. Additionally, inpatient compliance in the data set is a best estimate given variations in timing of when a patient leaves the operating room and how many doses of VTE chemoprophylaxis would be appropriate. Furthermore, variations in the documentation of hospital discharge time further complicate estimations of VTE chemoprophylaxis adherence on the last day of a hospital stay.

## Conclusions

This cohort study found that the overall VTE rate after major cancer surgery in the VHA was low. Inpatient chemoprophylaxis administration rates were high, and postdischarge VTE chemoprophylaxis prescribing was similar to that in other non-VHA health systems. Specialty and procedure site variation existed for chemoprophylaxis and may be justified given low risks of overall and postdischarge VTE. Continued development of population-specific and procedure site–specific risk calculators to guide VTE chemoprophylaxis is warranted.

## Notes

Supplement 1. **eTable 1.** VASQIP Cohort *Current Procedural Terminology* Codes for General Surgery **eTable 2.** VASQIP Cohort *Current Procedural Terminology* Codes for Thoracic Surgery **eTable 3.** VASQIP Cohort *Current Procedural Terminology* Codes for Urology **eTable 4.** VASQIP Cohort Venous Thromboembolism Rates by Procedure Site and Post-Operative Length of Stay

Supplement 2.
Data Sharing Statement
