# Supplementary material for: Venous Thromboembolism Chemoprophylaxis Adherence Rates After Major Cancer Surgery
Source: JAMA Netw Open. 2023 Sep 28;6(9):e2335311. doi: 10.1001/jamanetworkopen.2023.35311 (PMC10539988; doi:10.1001/jamanetworkopen.2023.35311)
Supplement: Supplement 1. — eTable 1. VASQIP Cohort Current Procedural Terminology Codes for General Surgery eTable 2. VASQIP Cohort Current Procedural Terminology Codes for Thoracic Surgery eTable 3. VASQIP Cohort Current Procedural Terminology Codes for Urology eTable 4. VASQIP Cohort Venous Thromboembolism Rates by Procedure Site and Post-Operative Length of Stay [file jamanetwopen-e2335311-s001.pdf]

Supplemental Online Content

Logan CD, Hudnall MT, Schlick CJR, et al. Venous thromboembolism chemoprophylaxis adherence rates after major cancer surgery. *JAMA Network Open.* 2023;6(9):e2335311 doi:10.1001/jamanetworkopen.2023.35311

eTable 1. VASQIP Cohort *Current Procedural Terminology* Codes for General Surgery

eTable 2. VASQIP Cohort *Current Procedural Terminology* Codes for Thoracic Surgery

eTable 3. VASQIP Cohort *Current Procedural Terminology* Codes for Urology

eTable 4. VASQIP Cohort Venous Thromboembolism Rates by Procedure Site and Post-Operative Length of Stay

This supplemental material has been provided by the authors to give readers additional information about their work.

© 2023 Logan CD et al. *JAMA Network Open*

eTable 1. VTE VASQIP Cohort Current Procedural Terminology Codes for General Surgery

Current Procedural Terminology	Freq.	Percent	Cum.
38100	1	0.01	0.01
43107	75	0.73	0.74
43108	2	0.02	0.76
43112	9	0.09	0.84
43113	1	0.01	0.85
43116	2	0.02	0.87
43117	53	0.51	1.39
43118	3	0.03	1.42
43121	1	0.01	1.43
43122	20	0.19	1.62
43620	18	0.17	1.80
43621	82	0.80	2.59
43622	5	0.05	2.64
43631	37	0.36	3.00
43632	95	0.92	3.92
43633	100	0.97	4.89
43634	2	0.02	4.91
43640	1	0.01	4.92
43644	6	0.06	4.98
43645	1	0.01	4.99
43652	1	0.01	5.00
43653	1	0.01	5.01
43659	60	0.58	5.59
44120	105	1.02	6.61
44125	6	0.06	6.67
44140	1,602	15.55	22.22
44141	99	0.96	23.18
44143	195	1.89	25.08
44144	104	1.01	26.08
44145	417	4.05	30.13
44146	196	1.90	32.04
44147	26	0.25	32.29
44150	62	0.60	32.89
44151	2	0.02	32.91
44155	19	0.18	33.09
44156	2	0.02	33.11
44157	14	0.14	33.25
44158	3	0.03	33.28
44160	649	6.30	39.58
44180	5	0.05	39.63
44186	20	0.19	39.82
44187	41	0.40	40.22
44188	152	1.48	41.69
44204	1,919	18.63	60.32
44205	886	8.60	68.93
44206	76	0.74	69.66
44207	484	4.70	74.36
44208	180	1.75	76.11

© 2023 Logan CD et al. *JAMA Network Open*

44210	34	0.33	76.44
44211	20	0.19	76.63
44212	15	0.15	76.78
44310	28	0.27	77.05
44312	1	0.01	77.06
44316	1	0.01	77.07
44320	78	0.76	77.83
44322	2	0.02	77.85
44950	8	0.08	77.92
44960	1	0.01	77.93
44970	31	0.30	78.24
45110	239	2.32	80.56
45111	31	0.30	80.86
45112	23	0.22	81.08
45113	8	0.08	81.16
45119	10	0.10	81.25
45121	1	0.01	81.26
45123	3	0.03	81.29
45126	6	0.06	81.35
45395	136	1.32	82.67
45397	27	0.26	82.93
47120	581	5.64	88.57
47122	45	0.44	89.01
47125	41	0.40	89.41
47130	87	0.84	90.25
47379	128	1.24	91.50
47562	43	0.42	91.91
47563	3	0.03	91.94
47600	36	0.35	92.29
47605	1	0.01	92.30
47610	1	0.01	92.31
48120	5	0.05	92.36
48140	219	2.13	94.49
48145	9	0.09	94.57
48146	5	0.05	94.62
48150	420	4.08	98.70
48152	23	0.22	98.92
48153	88	0.85	99.78
48154	11	0.11	99.88
48155	12	0.12	100.00
Total	10,301	100.00	

© 2023 Logan CD et al. *JAMA Network Open*

eTable 2. VTE VASQIP Cohort Current Procedural Terminology Codes for Thoracic Surgery.

Current Procedural Terminology	Freq.	Percent	Cum.
32440	102	3.85	3.85
32442	1	0.04	3.89
32445	5	0.19	4.08
32480	1,887	71.23	75.31
32482	97	3.66	78.97
32484	143	5.40	84.37
32486	38	1.43	85.81
32488	9	0.34	86.15
43107	84	3.17	89.32
43108	3	0.11	89.43
43112	22	0.83	90.26
43117	223	8.42	98.68
43118	8	0.30	98.98
43121	5	0.19	99.17
43122	14	0.53	99.70
43621	5	0.19	99.89
43632	2	0.08	99.96
43653	1	0.04	100.00
Total	2,649	100.00	

© 2023 Logan CD et al. *JAMA Network Open*

eTable 3. VTE VASQIP Cohort Current Procedural Terminology Codes for Urology.

Current Procedural Terminology	Freq.	Percent	Cum.
50220	278	1.63	1.63
50225	4	0.02	1.65
50230	419	2.45	4.10
50234	48	0.28	4.38
50236	18	0.11	4.49
50240	814	4.76	9.25
50543	1,331	7.79	17.04
50544	1	0.01	17.05
50545	1,041	6.09	23.14
50546	476	2.79	25.92
50548	173	1.01	26.94
51550	30	0.18	27.11
51555	6	0.04	27.15
51565	12	0.07	27.22
51570	14	0.08	27.30
51575	33	0.19	27.49
51580	18	0.11	27.60
51585	12	0.07	27.67
51590	109	0.64	28.30
51595	760	4.45	32.75
51596	112	0.66	33.41
51597	23	0.13	33.54
52601	535	3.13	36.67
52630	17	0.10	36.77
55801	4	0.02	36.80
55810	35	0.20	37.00
55812	11	0.06	37.06
55815	13	0.08	37.14
55821	37	0.22	37.36
55831	9	0.05	37.41
55840	391	2.29	39.70
55842	457	2.67	42.37
55845	1,646	9.63	52.00
55860	1	0.01	52.01
55862	16	0.09	52.10
55865	10	0.06	52.16
55866	8,175	47.84	100.00
Total	17,089	100.00	

© 2023 Logan CD et al. *JAMA Network Open*

eTable 4. Nationwide VHA Cohort Venous Thromboembolism Rates by Procedure Site and Post-Operative Length of Stay

	VTE Overall		VTE Inpatient		VTE Post-discharge		Post-op LOS (days)	
**Procedure Site**	**n**	**%**	**n**	**%**	**n**	**%**	**Median**	**IQR**
Colorectal (n=7,968)	107	1.3	70	0.88	37	0.81	6	4-8
Hepatobiliary (n=966)	14	1.4	8	0.83	6	0.2	6	4-8
Pancreas (n=792)	23	2.9	19	2.4	5	0.27	9	7-16
Esophagogastric (n=575)	15	2.6	13	2.3	3	0.0	9	7-15
Esophagus (n=367)	11	3.0	10	2.7	1	0.0	12	9-18
Lung (n=2,282)	25	1.1	14	0.61	11	1.0	7	5-9
Kidney (n=4,603)	39	0.85	21	0.46	18	0.66	3	2-4
Bladder (n=1,129)	29	2.6	19	1.7	10	0.93	7	6-11
Prostate (n=11,357)	122	1.1	25	0.22	97	1.3	2	1-3
**Total (n=30,039)**	**385**	**1.3**	**199**	**0.66**	**186**	**0.62**	**4**	**2-7**

VHA = Veterans Health Administration
VTE = Venous Thromboembolism
LOS = Length-of-Stay
IQR = Interquartile Range

© 2023 Logan CD et al. *JAMA Network Open*
