# Supplementary material for: Venous Thromboembolism Chemoprophylaxis Adherence Rates After Major Cancer Surgery
Source: JAMA Netw Open. 2023 Sep 28;6(9):e2335311. doi: 10.1001/jamanetworkopen.2023.35311 (PMC10539988; doi:10.1001/jamanetworkopen.2023.35311)
Supplement: Supplement 2. — Data Sharing Statement [file jamanetwopen-e2335311-s002.pdf]

Data Sharing Statement

Logan. Venous Thromboembolism Chemoprophylaxis Adherence Rates After Major Cancer Surgery. *JAMA Netw Open*. Published September 28, 2023.
doi:10.1001/jamanetworkopen.2023.35311

Data
Data available: No

Additional Information
Explanation for why data not available: The VASQIP dataset is not for public use.
